# How to understand the results of studies of glutamine supplementation

**DOI:** 10.1186/s13054-015-1090-7

**Published:** 2015-11-03

**Authors:** Jan Wernerman

**Affiliations:** Karolinska University Hospital Huddinge & Karolinska Institutet, K32 14186 Stockholm, Sweden

## Abstract

The lack of understanding of the mechanisms behind possible beneficial and possible harmful effects of glutamine supplementation makes the design of interventional studies of glutamine supplementations difficult, perhaps even hazardous. What is the interventional target, and how might it relate to outcomes? Taking one step further and aggregating results from interventional studies into meta-analyses does not diminish the difficulties. Therefore, conducting basic research seems to be a better idea than groping in the dark and exposing patients to potential harm in this darkness.

## Introduction

Meta-analyses do not bring much clarity over the possible beneficial effect of glutamine supplementation to critically ill patients. This is related to five issues: (a) The patients with hypoglutaminemia, who are the ones who have an association between a possible glutamine shortage and unfavorable outcomes, have never been properly investigated. (b) The mechanism that associated hypoglutaminemia with enhanced morbidity and mortality is not known. (c) The artificial separation according to route of administration has not been very helpful in interpreting the results. (d) The variable doses of glutamine and the combination with other nutrients have further blurred the interpretation of results. Finally, (e) the absence of characterization of nutritional status of the patients studied and the variable time course of critical illness at supplementation also contribute to the difficulty to digest the results presented.

## Hypoglutaminemia

A situation in which mechanism of action is not understood makes the meta-analysis technique particularly hazardous. The impact of route of administration, dosing, combination with other nutrients, definition of shortage, and time course of treatment all call for subgroup analyses in which the individual studies in the end will stand quite alone with their specific treatment protocols. Meta-analyses may be a useful tool when the mechanism of action is known and the peculiarities of the individual studies can be evaluated in that context. When the mechanism is obscure, results become much more speculative, and the value of combining studies into a meta-analysis is discounted and may even become confusing.

Until today, only one study provides plasma glutamine concentration at the time of study start [1]. It is unfortunate that the study protocol did not select the patients with hypoglutaminemia to randomization. Another unfortunate factor is that the intervention was not confined to glutamine supplementation but also included omega-3 fatty acids, and therefore post hoc subgroup analyses of the hypoglutaminemic subject did not shed any light on the effect of glutamine supplementation in that particular group. So to summarize, the hypothesis that glutamine supplementation may be beneficial in critically ill patients with hypoglutaminemia is still not addressed.

## Mechanism of action

The failure to understand the mechanism that associates hypoglutaminemia with an unfavorable outcome in critical illness is a crucial issue. Much more effort should be spent on exploring the pathophysiology of hypoglutaminemia in critically ill subjects. Extrapolation of results from animal studies has not been very helpful. Patients with hyperglutaminemia obviously do not need supplementation. This is a small group within the critically ill patients, often associated with hepatic failure [2, 3]. This is not confined to patients with acute fulminant liver failure but also applies to a large fraction of the patients with chronic or acute-on-chronic liver failure. Hyperglutaminemia may also occur in other critically ill patients and is shown to be associated with an unfavorable outcome [3]. Also, a high, but normal, plasma glutamine concentration during glutamine supplementation is related to post-intensive care unit (post-ICU) mortality [4]. In this case, however, this was strongly related to discharge Sequential Organ Failure Assessment (SOFA) scoring, making interpretation difficult. Overall, the mechanism behind hypo- and hyperglutaminemia and the relation to outcome in critical illness need to be further explored before starting up new treatment studies or new meta-analyses of earlier studies with the limitations in patient characteristics outlined above.

## Route of administration

In studies of nutrition support in the critically ill, it is a conventional approach to separate studies according to the route of nutrient administration. The background is the belief that the enteral route is superior to the parenteral. A number of studies comparing the two routes of administration have been published over the years, and an even larger number of meta-analyses have tried to summarize the results. The main problem with the studies has been whether or not the compared patient groups have really been comparable in gastrointestinal function and in nutrition intake. A recent study, performed in a modern ICU setting, failed to demonstrate any clinically relevant difference in outcomes related to route of administration. So the question remains of whether a differentiation of route of administration is relevant for glutamine supplementation. Plasma concentration is affected differently related to the route of administration [5], but is that a sufficient rationale to include or exclude studies in a summary? Again, the absence of mechanistic knowledge is striking. The difference in effect sometimes claimed may well be attributable to the fact that patients possible to feed by the enteral route are different from the patients not possible to feed by the enteral route, even if their risk scoring is identical.

Conventional parenteral nutrition excludes glutamine because of the instability of crystalline glutamine in aqueous solution. It is only by providing glutamine as a dipeptide that the amino acid component of parenteral nutrition becomes complete. Therefore, supplementing parenteral nutrition to have a glutamine content equivalent to that of enteral nutrition is not a controversial issue and therefore should be excluded from the discussion of specific glutamine supplementation. Nobody has suggested that glutamine be excluded from nutrition products.

## Dosing of supplementation

The dosing in glutamine supplementation has been totally arbitrary, regardless of whether the target has been to obtain a “pharmacological” effect or to substitute a shortage. In either case, successful treatment in terms of “immune parameters” or plasma glutamine concentration has not been related to the dose given. In the only dose-finding study published in the critically ill, it was demonstrated that the plasma glutamine level of healthy subjects is always possible to reach, but with an individualized dose [6]. The rationale of high or low doses in virtually all publications stays with the authors and has not been objectively verified. Therefore, separating high and low doses in a meta-analysis without better subject characterization does not seem to be a meaningful exercise.

## Nutritional status

Although the effect of nutrition is most likely related to nutritional status of the subjects receiving treatment, it is rare to find a nutritional characterization of included critically ill subjects in nutritional studies, beyond body mass index (BMI). There are observational reports that low and high BMI are risk factors for an unfavorable outcome in the critically ill [7]. This may be a reflection of the fact that total muscle mass is a predictor for outcomes, as suggested from radiologic estimations of muscle mass [8]. Nutritional characterization of included subjects is, of course, necessary in nutritional studies, and if subjects at a nutritional risk are systematically excluded, the external validity of result is correspondingly limited.

## Conclusions

To summarize, to substitute knowledge of the underlying mechanisms affected by an intervention, and thereby knowledge of the patients most likely to benefit from the intervention, by integrating studies with insufficient characterization of treatment and subjects into a meta-analysis will not expand knowledge. It may be hypothesis-generating if, by luck, relevant elements of the mechanism were included in the patient characterization. At a time when the underlying mechanism is not known, conducting basic research seems to be a much better idea than groping in the dark and exposing patients to potential harm in this darkness.

## Abbreviations

BMI: Body mass index
ICU: Intensive care unit
